# Commentary: Fostering nursing innovation to prevent and control antimicrobial resistance using approaches from the arts and humanities

**DOI:** 10.1177/1744987120914761

**Published:** 2020-05-03

**Authors:** Annie Topping

**Affiliations:** Professor of Nursing, University of Birmingham and University Hospitals Birmingham NHS Foundation Trust, Birmingham, UK

2020 has been designated the International Year of the Nurse and the Midwife marking the legacy established by Florence Nightingale and her bicentenary. It seems poignant that in 2020, despite all the advances in healthcare, we are facing a global pandemic caused by coronavirus, as well as probably as great if not a greater catastrophe, namely antimicrobial resistance (AMR). 2020 stands as a stark reminder of the fragility of the world health ecosystem and that communicable diseases remain a constant threat to global health and remain just as in Miss Nightingale’s day centre stage in healthcare practice. The World Health Organization (2015) highlighted six major factors contributing to AMR, and if not tackled, could kill millions by 2020. Although nurses may have little or no influence over one of the prime causes of AMR, the overuse of antibiotics in farming, we do have some control over the others. Namely, poor infection prevention and control (IPC) practice, on over-prescribing of antibiotics, ensuring patients are informed about the importance of completing any prescribed courses of antimicrobial treatment, and when we are responsible for administration or surveillance, ensuring doses of medication are given in a timely manner and not omitted.

Antibiotics were not even imagined in Florence Nightingale’s day but she recognised the relationship between infectious diseases and mortality, as illustrated in her iconic ‘coxcomb’ diagram showing the monthly death statistics among the Army of the East. If antibiotics had been available in Scutari many lives would no doubt have been saved. Yet her views on cleanliness and diet to prevent spread and recovery remain as significant today. It is probably unsurprising that many of the drawings produced by the participants in this study by Colin MacDuff and colleagues represent strategies to manage (un)cleanliness where nurses can, and where they have less control, often in peoples’ homes, the importance of hand washing. They also illustrate the inherent frustrations associated when trying to combat something that is invisible (pathogenic microorganisms) until harm is present and where the evidence of effective prevention is absent. I was minded of her words in *Notes on Nursing* when writing this commentary and reviewing the paper:

It cannot be necessary to tell a nurse that she should be clean, or that she should keep her patient clean, – seeing that the greater part of nursing consists in preserving cleanliness. (Nightingale, 1860: 87).

So to this interesting paper which outlines an approach designed specifically to foster the imagination and creativity of participants. This article only represents initial findings, and focusses on the discovery phase with data relating to practice ‘as is’ and the meaning of AMR to those involved. The researchers drew on methods of visualisation and co-design underpinned by the Double Diamond model developed by the UK Design Council (2015) to act as a way of conceptualising the process framing imagination and innovation in design. In the paper the authors focused on the discovery phase of the Double Diamond (discover, define, develop and deliver) model. The authors position the study theoretically within Sullivan’s (2005) dimensions of visualisation and normalisation process theory (May and Finch, 2009). They should be applauded for grappling with theory, particularly as it may serve to make any sense-making they employ more transparent, and ultimately findings more transferable. This is particularly significant as they entered the field with the objective to comprehend how nurses understood AMR and ‘AMR work’ and how nurses differentiated between AMR and IPC. They also sought to recruit a heterogeneous group of nurses to the two workshops that represented different settings, experience and level of specialisation, and had to limit the recruitment of IPC specialists who volunteered. This is significant at two levels: first if nurses are truly to contribute to antibiotic stewardship (Castro-Sanchez et al., 2019) it will be universally irrespective of role and context. Second, the researchers found that IPC specialists connected AMR, hygiene and IPC most clearly evident through the story-boarding activity they used. It is clear from the evidence that a policy driven top-down approach may not have the greatest influence in terms of mobilising the agency of nurses in addressing AMR. Yet most learning, as described in this paper, seemed to come from IPC specialist to ‘generalist’ nurse participants and not vice versa. This may as be my reading but begs questions about onward transfer of accepted reality (Collins and Stockton, 2018), the ownership of knowledge and work (whose role is AMR and/or IPC) and the problematic tension with specialisation within nursing and healthcare. I hope that this does not threaten any important contribution nurses can make to global health through antimicrobial stewardship.
